# Pigment dispersion syndrome: An atypical presentation

**DOI:** 10.4103/0974-620X.60022

**Published:** 2010

**Authors:** S. Bhallil, AI. Benatyia, B. El--Mahjoubi, O. El-Abdouni, H. Tahri

**Affiliations:** Department of Ophthalmology, University Hospital, Hassan II Fez, Morocco

Pigment dispersion syndrome (PDS) occurs most commonly in middle-aged myopic patients and is characterized by concentric iris pigmentation, iris transillumination defects, backbowing, Krukenberg spindle, and dense pigment deposits in the trabecular meshwork.[1] It can lead to elevated intraocular pressure (IOP) and glaucoma.[1] We describe a case of atypical PDS in a 30-year-old man with hypermetropia of +2.5 diopters.

**Figure 1:**
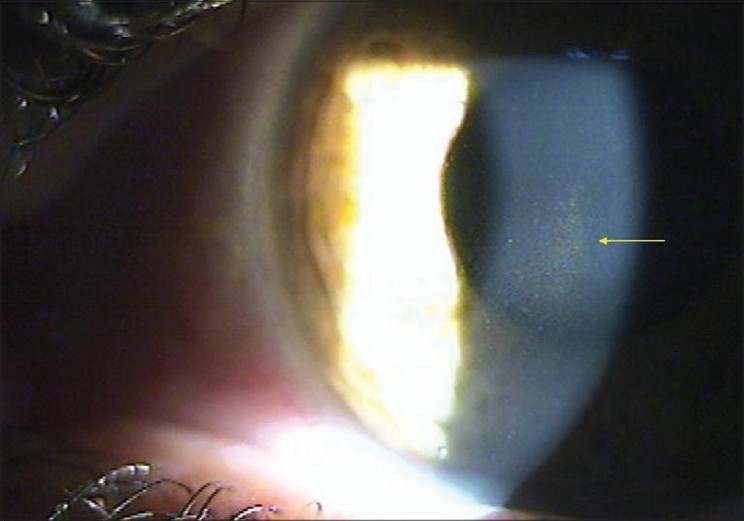
Krukenberg spindle is a spindle-shaped, vertical deposit of chocolate-brown colored pigment in the cornea (arrow)

**Figure 2:**
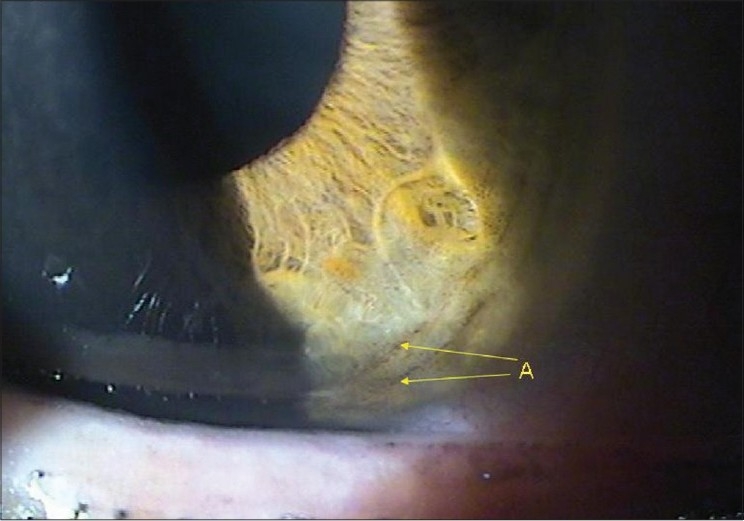
Slit-lamp examination revealed concentric iris pigmentation (arrows)

**Figure 3:**
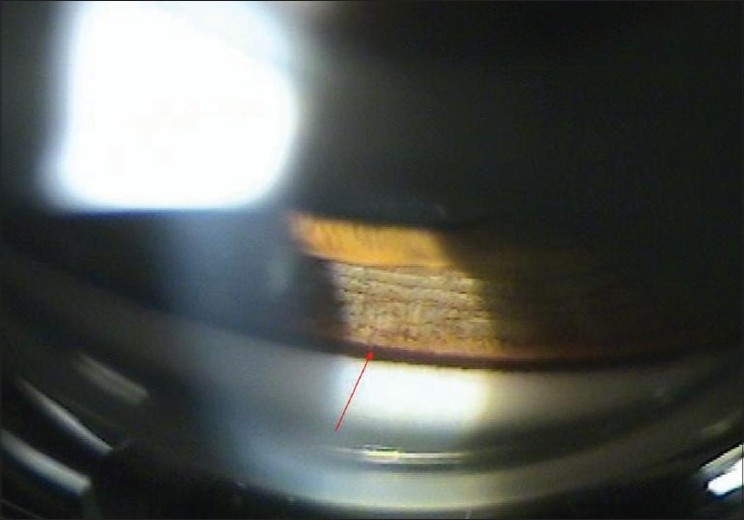
Gonioscopy revealed dense pigment deposits in the trabecular meshwork (arrow)

A 30-year-old man presented with asymmetric pigment dispersion resulting in pigmentary glaucoma in the right eye. There was no family history of glaucoma. On examination, the patient′s uncorrected visual acuity was 20/25 in the right eye (OD) and 20/20 in the left eye (OS). Refraction revealed hypermetropia of +2.5 diopters. Intraocular pressure was 38 mmHg OD and 16 OS. Both corneas were clear. Corneal pachymetry was 520 µm OD and 530 µm OS. Slit-lamp examination revealed Krukenberg spindle [Figure 1] with concentric iris pigmentation [Figure 2]. We noted absence of iris transillumination defects. Gonioscopy revealed heavy pigmentation of the trabecular meshwork [Figure 3]. The optic disk appearances and visual fields were within normal limits.

PDS results from iridozonular friction and some possible contribution from iridociliary process contact with resultant liberation of pigment from the posterior iris. In majority of the patients with PDS, there is a concave approach of the iris as it inserts into the anterior chamber angle.[1,2] The cause of this concavity remains unclear.[1] The liberated pigment accumulates on the corneal endothelium, the front surface and the circumferential furrows of the iris, the posterior lens capsule and lens zonules within the trabecular meshwork. This pigment dispersion is mainly due to aqueous convection currents.[1,2] When aqueous outflow is affected and intraocular pressure rises, nerve damage may ensue. This process is known as pigmentary glaucoma (PG). Pigment dispersion syndrome has been found most commonly in patients between the ages of 30 and 50 years.[3] Myopia is frequently associated with pigment dispersion syndrome with a mean myopic correction of-3.9 diopters.[3] Hypermetropia with PDS has been rarely reported.[4]

The risk of developing pigmentary glaucoma from pigment dispersion syndrome is about 10% in most series. Young, myopic men were most likely to have pigmentary glaucoma.[3] An IOP greater than 21 mmHg at initial examination is usually associated with an increased risk of conversion.[3] Possibly due to a thicker iris stroma, the iris transillumination defects will rarely be present in patients of African descent.[1] If medical (Beta-adrenergic antagonists, Parasympathomimetics, Alpha-adrenergic agonists, Carbonic anhydrase inhibitor, Prostaglandin analogs) and laser (Laser trabeculoplasty, Laser iridectomy) therapy fail to control the IOP in PG, then glaucoma-filtering surgery is an option.[1]
